# Involvement of microRNA-Mediated Gene Expression Regulation in the Pathological Development of Stem Canker Disease in *Populus trichocarpa*

**DOI:** 10.1371/journal.pone.0044968

**Published:** 2012-09-18

**Authors:** Jia-Ping Zhao, Xiao-Ling Jiang, Bing-Yu Zhang, Xiao-Hua Su

**Affiliations:** 1 Key Laboratory for Silviculture and Conservation, Ministry of Education; College of Forestry, Beijing Forestry University, Beijing, People’s Republic of China; 2 State Key Laboratory of Forest Genetics and Breeding; Institute of New Forestry Technology, Chinese Academy of Forestry, Beijing, China; 3 College of Forestry, Agricultural University of Hebei, Baoding, People’s Republic of China; 4 State Key Laboratory of Tree Genetics and Breeding; Research Institute of Forestry, Chinese Academy of Forestry, Beijing, People’s Republic of China; Soonchunhyang University, Republic of Korea

## Abstract

MicroRNAs (miRNAs), a type of short (21–23 nucleotides), non-coding RNA molecule, mediate repressive gene regulation through RNA silencing at the post-transcriptional level, and play an important role in defense and response to abiotic and biotic stresses. In the present study, Affymetrix® miRNA Array, real-time quantitative PCR (qPCR) for miRNAs and their targets, and miRNA promoter analysis were used to validate the gene expression patterns of miRNAs in *Populus trichocarpa* plantlets induced with the poplar stem canker pathogen, *Botryosphaeria dothidea*. Twelve miRNAs (miR156, miR159, miR160, miR164, miR166, miR168, miR172, miR319, miR398, miR408, miR1448, and miR1450) were upregulated in the stem bark of *P. trichocarpa*, but no downregulated miRNAs were found. Based on analysis of the miRNAs and their targets, a potential co-regulatory network was developed to describe post-transcriptional regulation in the pathological development of poplar stem canker. There was highly complex cross-talk between diverse miRNA pathway responses to biotic and abiotic stresses. The results suggest that miR156 is probably an integral component of the miRNA response to all environmental stresses in plants. Cis-regulatory elements were binding sites for the transcription factors (TFs) on DNA. Promoter analysis revealed that TC-rich repeats and a W1-box motif were both tightly related disease response motifs in *Populus*. Promoter analysis and target analysis of miRNAs also revealed that some TFs regulate their activation/repression. Furthermore, a feedback regulatory network in the pathological development of poplar stem canker is provided. The results confirm that miRNA pathways regulate gene expression during the pathological development of plant disease, and provide new insights into understanding the onset and development of poplar stem canker.

## Introduction

MicroRNAs (miRNAs) are short (21–23 nucleotides) non-coding RNA molecules that mediate repressive gene regulation through RNA silencing at the post-transcriptional level in plants and animals [1]. Besides their contribution to plant growth, development, and metabolism, miRNAs are also integral components of plant responses to adverse environmental conditions [1], [2], [3]. Increasing evidence indicates that miRNAs are involved in the gene expression regulatory response to abiotic stresses in plants, such as low and/or high temperature [4], [5], [6], water [4], [6], salt [4], [6], [7], [8], heavy metal [8], oxygen [8], [9], UV-B light [10], [11], mechanical tresses [12], and hormone [7]. Plant miRNAs also regulate gene expression under biotic stresses. Research on miRNAs mediating gene expression in virus-plant interactions has been reviewed [13]; however, there is little information on miRNAs induced by fungal or bacterial pathogens.

*Arabidopsis* miR393 was the first miRNA implicated in plant defense to bacterial pathogens, presumably by repressing auxin signaling, and contributes to resistance against virulent *Pseudomonas syringae* pv. tomato strain DC3000 (Pto DC3000), non-pathogenic *P. fluorescens* and *Escherichia coli*, implying that miR393 is a key component of plant basal defense [14], [15]. In addition, miR398 was found to be downregulated in response to biotic stress in *Arabidopsis* [7]. After inoculation of *Arabidopsis* with avirulent Pto DC3000 strain (avrRpm1 or avrRpt2), expression of miR398 decreased, while expression of its target gene (Cu/Zn superoxide dismutase 1, CSD1) increased in leaves [7]. The response of CSD to abiotic and biotic stresses indicates that downregulation of miR398 provides a molecular indicator of disease resistance in plants. Additionally, it was reported that the expression level of tomato (*Solanum lycopersicum*) miR482 suppressed at 4 h after inoculation by bacterial (Pto DC3000 and hrcC) or viral pathogens, while the expression level of their target genes (Nucleotide Binding Site–Leucine-Rich Repeats proteins coding zgenes, NBS-LRR protein coding genes) increased [16]. These studies suggested some miRNAs that target disease resistence genes could play an important role in the defense against pathogen attack.

It is known that miRNAs also mediate gene expression after fungal pathogen attack. Lu et al. (2007) cloned and identified 26 miRNAs from stem xylem of loblolly pine (*Pinus taeda*) that respond to the fusiform rust pathogen (an endemic rust fungus, *Cronartium quercuum* f. sp. *fusiforme*), and also predicted 82 plant disease-related transcripts that may also respond to miRNA-guided regulation in development of fusiform rust galls [17]. Downregulation of 10 out of 11 large miRNA families of loblolly pine - including seven pine-specific families - was also observed in galls induced by *C. quercuum* [17]. These results reveal a new genetic basis for host-pathogen interactions in plants. Xin et al. (2010) used Solexa high-throughput sequencing to clone 153 miRNAs from wheat leaves infected with the common strain of powdery mildew (*Erysiphe graminis* f. sp. *tritici*) or following heat stress treatment. Among these, 24 and 12 miRNAs were responsive to powdery mildew infection and heat stress, respectively, indicating that diverse miRNAs are responsive to infection and stress and function in plant responses to both biotic and abiotic stresses [5].

Plant miRNAs are not only involved in plant-pathogen interactions, but also participate in the regulation of symbiotic interactions between plants and microorganisms. Specific miRNAs have been demonstrated to mediate post-transcriptional regulation in roots containing the legume nitrogen-fixing rhizobia symbiotic system [18], [19]. Gu et al. (2010) also revealed the role of miRNAs in the establishment of tomato-arbuscular mycorrhizal (AM) symbiosis. In this system, conserved miRNAs are differentially regulated by phosphate and/or AM symbiosis, paving the way for further characterization of the functions and signaling pathways in AM symbiosis [20].

Trees of the genus *Populus* (e.g., aspen, poplar, cottonwoods) found in the Northern hemisphere are model organisms for the study of perennial woody plants, and valuable economic and ecological resources. With the rapid increasing of poplar plantations, poplar blister canker, a stem bark disease caused by an extremely polyphagous fungus (*Botryosphaeria dothidea*) is becoming the most severe disease in China. Recently, many miRNAs responsive to abiotic stresses in *Populus* have been reported [4], [7], [10], [12], [21], [22]. Similar to the facts of miRNAs-mediated gene expression regulation involved in pathological development of wheat disease [5], pine disease [17], and tomato diseases [20], a specific miRNA expression profile is anticipated during the plant-pathogen interaction in *Populus*. However, the families of miRNAs, time of activation, and how they guide specific gene expression during poplar-*B. dothidea* or other poplar-pathogen interaction is still unreported.

For a better understanding of the miRNA-mediated gene expression regulation patterns of poplar canker, microarray analysis, real-time qPCR validation of miRNAs and their targets, and promoter analysis of responsive miRNAs in *P. trichocarpa* after *B.dothidea* inoculation were assessed. Environmental stresses, including potential plant diseases, are predicted to become more severe and widespread in the future. A better understanding of the mechanisms of miRNA-mediated pathogenesis in *Populus* should shed light on management strategies for tree diseases.

## Results

### The miRNA Microarray Revealed 41 Fungi-responsive miRNAs Genes in *P. trichocarpa*

**Figure 1:**
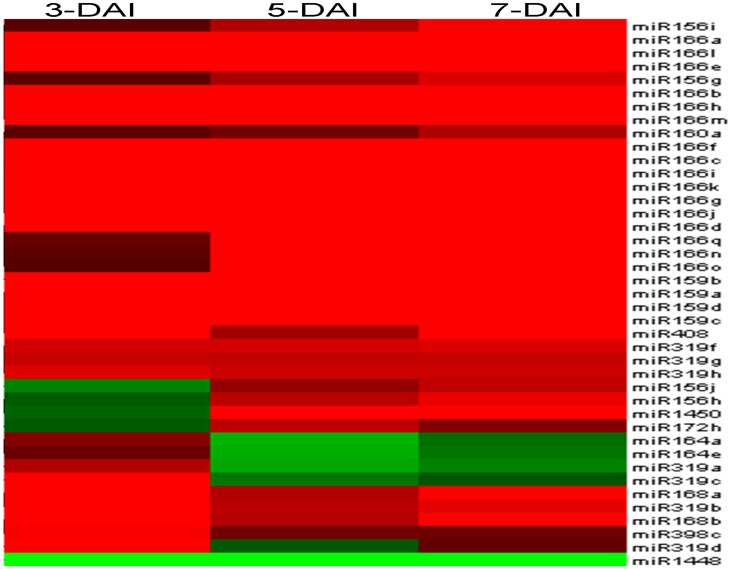
The fungi-responsive miRNAs of *P. trichocarpa* identified via microarray analysis. CK: inoculated with PDA after 3 days, control; 3 (and 5 and 7) DAI: inoculated with fungal pathogen after 3 (and 5 and 7) days; Gene Cluster 3.0 was used for clustering of differentially expressed miRNAs. Red indicates fungi-induced miRNAs, green indicates fungi-repressed miRNAs.

In ours previous inoculation study, we found that the symptoms of canker disease appeared on the bark of *P. trichocarpa* at 10–14 days after inoculation by canker pathogen, *B.dothidea* strain CZ060. And the expression of some thaumatin-like proteins (TLPs) coding genes (for example, gw1.IV.1632.1) were induced by *B.dothidea* strain CZ060 in the poplar bark at 2 days after inoculation (data unpublished). Then, to detect the expression patterns of miRNAs in the pathological development of poplar stem canker, the expression profiles of miRNAs in *P. trichocarpa* bark were detected 3, 5, and 7 days after inoculation (DAI) and compared to the control using the Affymetrix® miRNA Array GeneChip. The Affymetrix genechip produced 234 known poplar miRNA probes belonging to 42 miRNAs gene families. A total of 41 probes with significantly altered expression were detected in at least one treatment. These probes belong to 12 miRNA families (miR156, miR159, miR160, miR164, miR166, miR168, miR172, miR319, miR398, miR408, miR1448, and miR1450) and account for 17.52% of the 234 probes. A total of 38 of the 41 miRNA probes were upregulated and 3 were downregulated after fungal challenge (Table S1) (Figure 1). However, a large number of poplar miRNAs (193 of 234 miRNAs) were not differentially regulated or response to pathogen inoculation at these three time points. These results provided the first experimental evidence that a set of miRNAs are involved in the regulation of gene expression in the pathological development of poplar canker disease.

### Upregulated Expression in 12 Fungi-response miRNA Families

**Figure 2:**
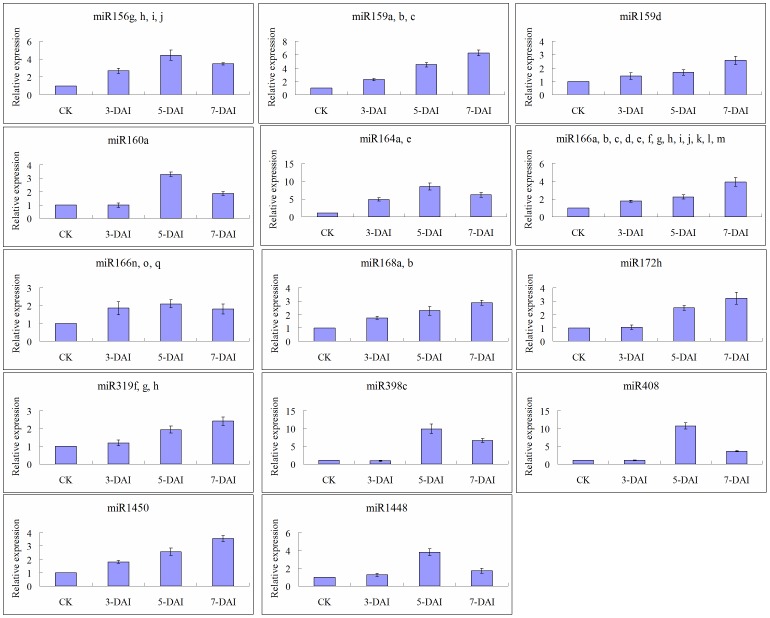
Real-time qPCR validation of the fungi-responsive miRNAs in bark of *P. trichocarpa*. Levels of miRNA were quantified in total RNA isolated from plants inoculated with fungal pathogen at 3, 5, and 7-DAI, and the corresponding control (CK), using quantitative real-time PCR, and were normalized to the level of 5.8S rRNA in the sample. Error bars represent standard deviations of three PCR replicates of a single reverse transcription reaction. The normalized miRNA levels in CK were arbitrarily set to 1.

Fifteen qPCRs were performed to validate the expression of 12 fungi-responsive miRNA families; the results are presented in Table S2 and Figure 2. A total of 14 qPCRs were analyzed; data for miR319A (designed for detecting expression of miR319a-d) were omitted for the control. The miRNAs detected in 14 reactions were upregulated at 3, 5, and 7 DAI in *P. trichocarpa*, with two exceptions: miR398 and miR160 at 3 DAI. According to the miRNA expression patterns after real-time qPCR analysis, these 12 miRNAs can be divided into two groups. Group I included miR159, miR168, miR172, miR319 (miR319f-h), miR1450, and 13 members of miR166. The miRNAs of this group continuously increased at 3, 5, and 7 DAI. Group II contained miR156, miR160, miR164, miR1448, miR398, miR408, and three members of miR166; expression of these miRNAs was always higher at 5 DAI than that at 3 or 7 DAI.

Most real-time qPCR results were consistent with the results of the microarray analysis, with the exceptions of miR164a, miR164e, and miR1448. As shown in Figure 1, reduced expression was detected in these three miRNAs in microarray analysis, whereas they increased in qPCR validation analysis. Because real-time qPCR is considered the gold standard for validation of gene expression, we considered that 12 fungi-responsive miRNAs shared upregulation at 3 to 7 DAI.

### Diverse Functions of the Target Genes of the Fungi-responsive miRNAs

The target genes of poplar miRNAs were identified by either bioinformatics or experimental approaches [4], [12]. The corresponding target genes of the 12 fungi-responsive miRNAs and their functions are listed in Table S3. The functions of these target genes were diverse, and they were involved in a broad spectrum of cellular processes, including defense, development, metabolism, transportation, cytoskeleton construction, signal transduction, and programmed cell death (PCD). Based on the functional descriptions, the predicted target genes can be grouped into three major categories. The first category includes genes involved in defense response. For example, the target genes of miR156 and miR1448 encoded plant disease resistance proteins (NBS-LRR proteins); two targets of miR164 encoded LRR defense proteins and TMV-resistance proteins; one target of miR398 and miR159 encoded two pathogenesis-related proteins, CSD and peroxidase (POD), respectively; and one target of miR159 encoded cytokinin oxidase (CKX) family proteins. Although it is generally accepted that CKX play a major regulatory role in the determination of cytokinin levels in plants [23], one transcriptome analysis suggested that cytokinin oxidase/dehydrogenase overexpressing lines show resistance to clubroot disease in *Arabidopsis* [24]. Moreover, plastocyanin-like proteins (target of miR408) were found to be associated with PCD [25], plant defense responses [26], and pathogen infection [27]. Therefore, this group of miRNAs and their target genes might determine the resistance to fungal stress in *Populus*.

The second category of predicted targets was made up of genes that encode transcription factors (TFs) or are involved in signal cascade pathways. For instance, the targets of miR156 and miR160 were genes encoding squamosa promoter-binding (SPB) proteins; the target genes of miR166 encoded a homeodomain-leucine zipper protein (HD-ZIP); and miR159, miR164, and miR319 targeted myeloblastic leukemia (MYB) factors. The targets of miR156 and miR160 were genes encoding ARF, whereas miR164 and miR166 both targeted NAC domain proteins. By binding to their corresponding cis-regulatory elements in the DNA sequence, these TFs could regulate the expression of protein-coding genes downstream of cis-regulatory elements; these signal cascade proteins could also regulate signal transduction in plants. The third category of predicted targets was involved in metabolic pathways and diverse physiological processes. For example, the sole target of miR1450 encoded a leucine-rich repeat transmembrane protein kinase, and five targets of miR172 encoded a homeotic protein, APETALA2, that is involved in floral development. These results suggest that fungal attack altered the gene expression of not only resistance- and pathogenesis-related genes but also genes involved in some basic physiological processes. Moreover, one target of miR168 encoded an argonaute (AGO1) protein; by cleavage of the mRNA of AGO1, miRNA-mediated gene expression regulation in pathological development was feedback-regulated.

**Figure 3:**
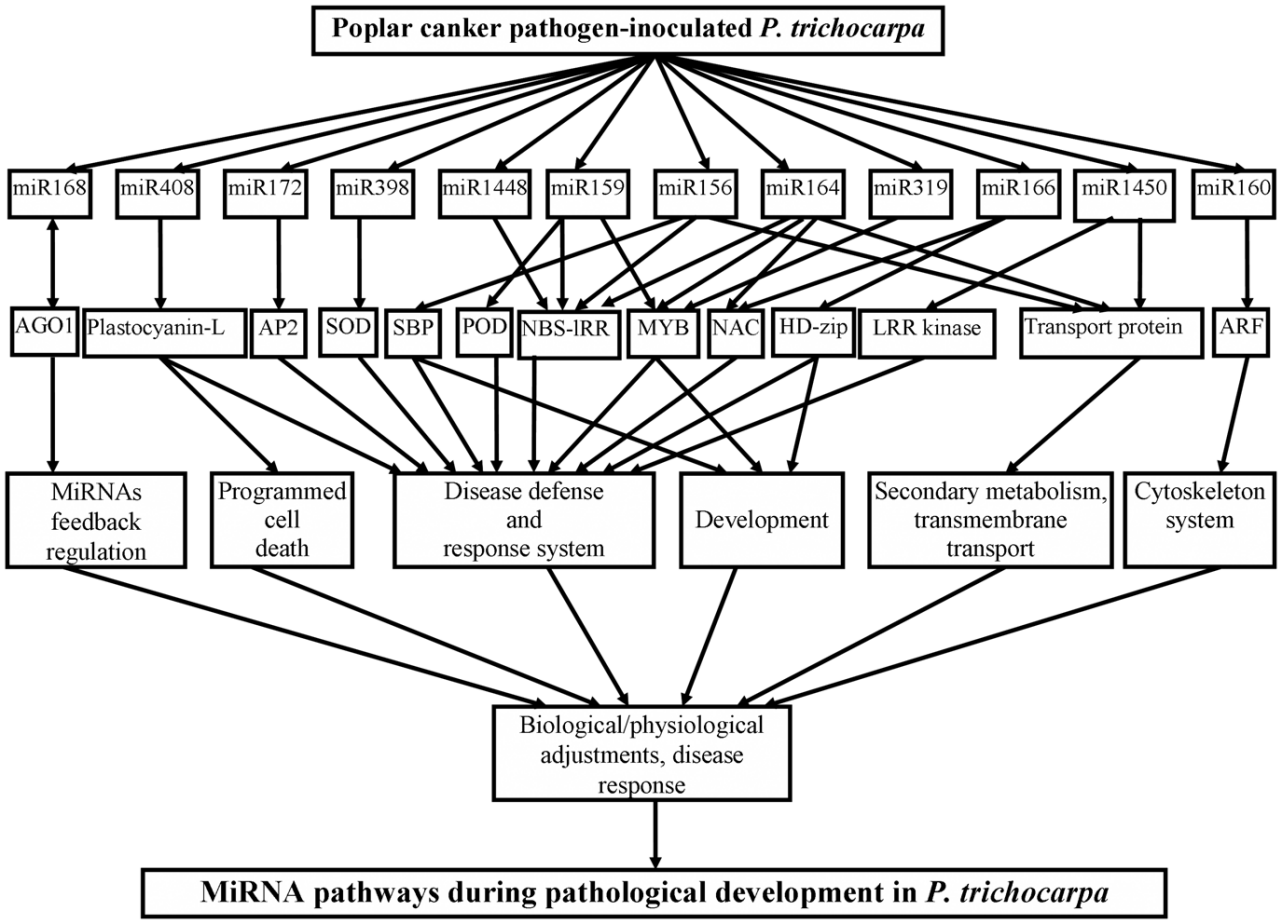
The potential regulatory pathways of fungi-responsive miRNAs in *P. trichocarpa*.

All fungi-responsive miRNAs targeted more than one gene, and each target gene was always involved in several physiological processes. Hence, gene expression in pathological development was always regulated through a complex network. These results, taken together with those of previous studies, indicate that there is an integrated co-regulatory network in the pathological process of poplar canker disease (Figure 3).

### Regulation of miRNA-mediated Gene Expression in the Pathological Development of Poplar Canker

**Figure 4:**
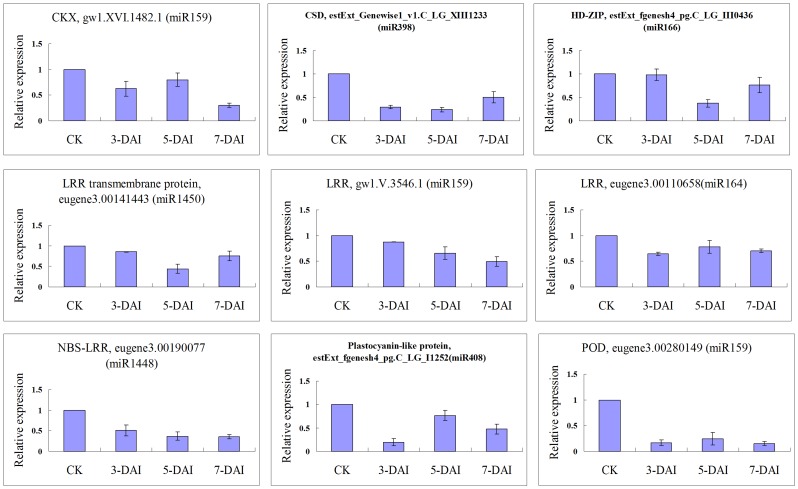
Real-time qPCR expression analysis of fungi-responsive miRNA targets. The mRNA level of CKK, CSD, HD-ZIP III protein, LRR, LRR transmembrane protein, NBS-LRR, Plastocyanin-like protein and POD were normalized to the internal reference UBQ and then to the level of the control. The presented data are the average of two independent experiments ± SD.

Nine target genes that encoded CSD (estExt_Genewise1_v1.C_LG_XIII1233, miR398), CKX (gw1.XVI.1482.1, miR159), HD-ZIP III protein (estExt_fgenesh4_pg.C_LG_III0436, miR166), LRR protein (gw1.V.3546.1, miR159; eugene3.00110658, miR164), LRR transmembrane protein (eugene3.00141443, miR1450), NBS-LRR protein (eugene3.00190077, miR1448), Plastocyanin-like protein (estExt_fgenesh4_pg.C_LG_I1252, miR408) and POD (eugene3.00280149, miR159) were selected to validate gene expression of the poplar stem canker-responsive miRNAs using real-time qPCR. As shown in Figure 4 and Table S4, these nine target genes always decreased at 3, 5, and 7 DAI in *P. trichocarpa* plantlets, although there was one exception at 3 DAI in the HD-ZIP III protein (the relative expression level was 0.98±0.12 when compared to the control). In general, if a miRNA is upregulated, its target genes are likely to be coherently downregulated post-transcriptionally. Therefore, we thought that these downregulated expression patterns of target gene were roughly corresponding to the upregulated expression patterns of miRNAs that validated by RT-qPCR method. However, there is an exception, for example, the accumulation of the miR1450 reached a maximum amount at 7 DAI, but the expression of its target (LRR transmenbrane protein gene) at 7 DAI is significant lower than that of 5 DAI. We inferred that the upregulated fungi-response miRNAs did decrease the expression of their target genes, which include not only the disease resistance and/or responsive genes, but also many basic metabolism-related genes described above.

In our preliminary experiments, the symptoms of bark necrosis (a typical symptom of canker disease) appeared around the inoculation sites on the stem bark of *P. trichocarpa* clone M16 10 days after inoculation with *B. dothidea* strain CZ060 (data not shown); that is, fungal inoculation induced poplar stem canker disease in *P. trichocarpa*. As the occurrence of symptoms were much later than the change in gene expression in the cells, the results in this study represents the regulatory expression of genes during the pathological development of stem canker. The decreased expression of fungi-responsive miRNAs targeted genes implies a decline in all cell functions (including disease resistance and other basic metabolic functions) in *P. trichocarpa*. Through the continuous and irreversible decline in cell functions, some cells invaded by canker pathogen lost their vitality, died, and necrosis symptoms appeared around the inoculation sites in the poplar stem bark after 10 or more days. Therefore, the upregulated expression patterns of all fungi-responsive miRNAs were the main characteristic of miRNA-mediated gene expression in the interaction between the susceptible poplar and canker pathogen. Interestingly, the decline also included the miRNA pathways themselves. For example, the upregulation of miR168 decreased the level of AGO1 protein, and consequently the production of all miRNAs was repressed.

We noticed that one newly study revealed that the expression of tomato miR482, that targeted some NBS-LRR protein genes, suppressed after inoculation by bacteria and virus pathogens [16]. But why the expression level of poplar miR1448 and miR164 which also targeted NBS-LRR protein genes increased response to canker pathogen? The difference might due to length of the inoculation time in the two studies. The tomato materials were collected at 4 h after inoculation while the poplar materials were collected at 3, 5 and 7 days after inoculation. Results in our study might represented the gene expression patterns of the pathological development of poplar stem canker disease, while results in tomato might represented the expression pattern of miRNAs at the beginning of bacterial or viral disease.

### Analysis of Cis-acting Elements of the Fungi-responsive miRNA Genes

To further elucidate the expression characteristics of fungi-responsive miRNAs, the distribution and occurrence of several known stress-responsive elements were analyzed, including the ABA-response (ABREs), anaerobic induction (AREs), heat stress-response (HSEs), low temperature-response (LTRs), MYB binding site involved in drought-induction (MBS), defense and stress-responsive elements (TC-rich repeats, ATTTTCTTCA), and fungal elicitor responsive elements (W1-box, TTGACC). For the natural linkage between phytohormones and pathological processes and/or resistance development, some other regulatory elements associated with plant hormones were also detected, such as MeJA-responsive (CGTCA motifs), ethylene-responsive (EREs), and gibberellin-responsive (GAREs, AAACAGA) elements, and low-temperature and gibberellin signals (P-box, CCTTTTG) (Table 1).

**Table 1:** Cis-acting elements of fungi-responsive miRNAs genes in *P. trichocarpa*.

Cis-element^1^	ARE	ABRE	box-W1	CGTCA-motif	ERE	GARE	HSE	LTR	MBS	P-box	TC rich
miR1448^2^	2	2		4	1		11		3		2
miR1450	2	1	3	1		1			2		1
miR156g		2	1	3		3			5		3
miR156h	2	2	2	3	1		1		3		3
miR156i	2		2						2		3
miR156j	3	2			1		3		2		
miR159a			1						1	1	2
miR159b	2	1	2	1			3	2	3	2	3
miR159c	1	1	1	1			1		1		2
miR159d	2	3	1	1		1	2	1	5	2	2
miR160a		2	1	2	1		3				2
miR164e	3	2	2	2			1		3		4
miR164a			1	2	1	1				1	3
miR166a	3	2		2	1	2	1			1	3
miR166b	1		1		1	2	2		1		2
miR166c		1	5		1	1	3				3
miR166d	4				2	1	6		1		2
miR166e	2	1	4		1	1		1	3	1	2
miR166f		6	1	3	1		5	1	1		5
miR166g	3	1	3			1	5	1	2	1	2
miR166h	2	2	1	1				1	2		3
miR166i		2	2	1					1		1
miR166j	1	1	1	2	2	2	1		2		2
miR166k	2	1	1	2	1	3	1		2		4
miR166l	1					3	2	1	1		5
miR166m	4	1	1	1		1	3	3	4		
miR166n	2		1	4		3	1		5	1	3
miR166o	2	2	2	2	1	1			1	1	5
miR166q		6	1	3	1		5	1	3		5
miR168a	3	4			1	1	3		1	1	4
miR168b	5	4	2				3		1		3
miR172h	2	1		2		1			2	1	3
miR319a	1	1	1		1	2	3	1	1	1	2
miR319b	3			3		1	2		1		4
miR319c	1			2		1	1				1
miR319d			1	1			3				4
miR319f	2		2				1	1	2		3
miR319g	2	1	1	3	2		2	1	1	2	6
miR319h	10		10	1			2	1	5		6
miR398c	2	2	1	1	1	1	4	1	1	2	5
miR408	8		1				1	1	7		
Meanin total miRNAs	2.1	1.4	1.5	1.4	0.5	0.8	2.1	0.4	1.9	0.4	2.9
Abundance (%)	78.0	68.3	82.9	68.3	46.3	51.2	75.6	31.7	85.4	34.2	92.7

Note: 1. Phytohormone and stress related cis-elements: ABA-responsive element (ABRE), anaerobic response element (ARE), ethylene responsive element (ERE), defense and stress responsiveness (TC-rich repeats), fungal elicitor responsive element (box-W1), gibberellin-responsive element (GARE), gibberellin-responsive element (P-box), heat stress element (HSE), low temperature responsive element (LTR), MeJA-responsiveness element (CGTCA-motif and TGACG-motif), MYB binding site (MBS). 2. For miR482 located on the 187bp before the mature sequence of miR1448 in *P.trichocarpa* LG_VIII, the promoter analysis of miR1448 was undertaken on the up-stream of pre-miR482 (2500bp in length).

TC-rich repeats, MBS, and W1-box were the three most prevalent cis-elements present in fungi-responsive miRNAs of *Populus*, accounting for 92.7%, 85.4%, and 82.9% of the 41 fungi-responsive miRNAs molecules, respectively. MBS is the binding site for MYB TFs. MYB is thought to take part in the complicated signaling network through which plants respond to changes in the surrounding environment [28], especially drought stress. Results of promoter analysis revealed that fungi-responsive miRNAs had characteristically high inducibility under various environmental stresses, especially fungal pathogen stress. In addition, the high occurrence of MBS may represent cross-talk between the poplar disease-response mechanism and the drought-response mechanism mediated by miRNAs.

Besides these three defense and/or response motifs, four hormone-related cis-regulatory elements (ERE, GARE, CGTCA-motif, and ABRE) and ARE also had higher ratios (from 46.3 to 78.0%) in all fungi-responsive miRNAs. Many studies have demonstrated that plant defense and response to disease are tightly associated with differential regulation of phytohormones [29]. Therefore, besides their direct regulation of plant disease resistance, phytohormones also negatively regulate resistance by binding to cis-elements of the fungi-responsive miRNAs in the plant-microorganism interaction. One high-temperature stress-related cis-element (HSE) was found in 75.6% of the 41 fungi-responsive miRNAs, while two low-temperature-related motifs (LTR and P-box) with the lowest distribution ratios accounting for 31.7% and 34.2% of all miRNAs. This result might imply that the *P. trichocarpa* response mechanism to high temperature shares aspects with the pathogen defense response or responsive mechanism to low temperature.

## Discussion

### A Subset of miRNAs are Involved in Poplar Response to Fungal Pathogen Stress

**Figure 5:**
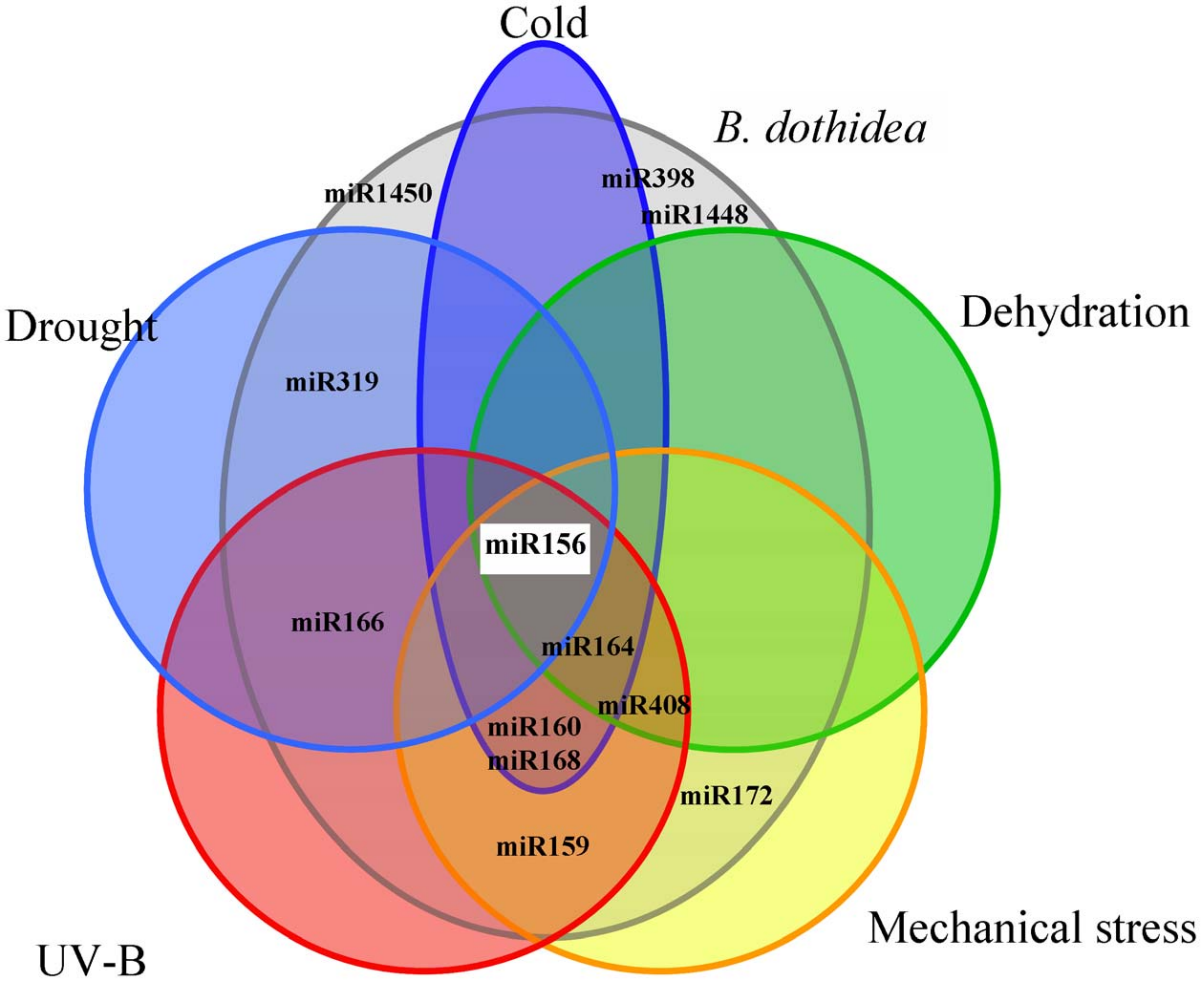
Cross-talk in miRNA pathways of *Populus* in response to different biotic and abiotic stresses. Nine of 12 fungi-response miRNAs of *P. trichocarpa* also involved in the response to one or more kinds of abiotic stresses: cold stress (Lu *et al.*, 2008), dehydration (Li *et al.*, 2009), drought (Li *et al.*, 2011), mechanical stress (Lu *et al.*, 2005) and UV-B light (Jia et al., 2009) in other species of *Populus*. Among those miRNAs, miR156 responded to all stresses, suggesting it might be an integral component of miRNA pathways for all biotic and abiotic stresses in *Populus*.

We found 12 miRNAs families involved in poplar response to fungal pathogen stress. Among these miRNAs, several also respond to pathogenic fungi in other plants, for example, miR156, miR159, miR160, and miR319 in galled loblolly pine stem infected with *C. quercuum* f. sp. *fusiforme*, miR156, miR159, miR164, and miR396 in wheat leaves infected with two powdery mildew pathogens of *E. graminis* f. sp. *tritici* [5]. Interestingly, several fungi responsive-miRNAs found in this study have also been reported to be involved in the response of *Populus* to the abiotic stresses in previous work. For example, expression of miR156, miR160, miR164, miR166, miR168, miR398, and miR408 increases while miR159 shows reduced expression after UV-B radiation in *P. tremula* [10]. MiR156, miR159, miR160, miR164, miR168, miR172, and miR408 are significantly expressed in tension- and compression-stressed developing xylem of *P. trichocarpa* [12], and miR156, miR160, miR164, and miR168 also respond to cold stress in *P. trichocarpa* [4]. In *P. euphratica*, miR156, miR164, and miR408 are differentially expressed under dehydration stress [22], and miR156, miR319, and miR166 respond to drought stress [21]. Taken together, these results indicate that cross-talk exists between miRNA pathways for fungal pathogens and abiotic stress responses (UV-B light, mechanical, cold, dehydration, and drought stress) in *Populus* (Figure 5). These complex regulatory networks of miRNAs could contribute to the ability of poplar to survive an ever-changing and often stressful environment.

### The miR156 is an Integral Component of miRNA Pathways for all Biotic and Abiotic Stresses in Plants

The most interesting discovery in this study was that miR156 responded to all six of the above stresses in *Populus*, although the expression patterns differed in response to cold, mechanical, drought, dehydration, and fungal invasion stresses. In other plants, miR156 was also reported to respond to several kinds of biotic and abiotic stress. The level of miR156 significantly decreases/increases in phosphate-deprived and temperature-stressed *Arabidopsis* [30], [31], in dehydration-stressed *Hordeum vulgare* (barley) [32], in drought-challenged rice [33], in domesticated durum wheat (*Triticum turgidum* ssp. *durum*) [34], and in response to different abiotic stresses in tobacco [35]. In addition, miR156 is significantly repressed in galled loblolly pine stems infected with the fungus *C. quercuum* f. sp. *fusiforme* [17] and in powdery mildew pathogen-stressed wheat [5]. Moreover, viruses or viral suppressors also switch on the expression of tobacco miR156 [36] and *Arabidopsis* miR156 [37]. In addition, miR156 is considered an evolutionarily conserved regulator of vegetative phase change in both annual herbaceous plants and perennial trees [38], accompanied by miR172, where miR156 controls the transition from juvenile to adult development [39], [40]. Moreover, 172 miR156 molecules found in 24 species, both gymnosperms and angiosperms, are in release 18 of the miRBase (http://www.mirbase.org). The ubiquitous distribution, evolutionary conservation, and crucial roles in biological rhythms suggest that miR156 might play a key role in the transition between differential physiological stages or phases. Together, we infer that miR156 could be an integral component of the miRNA-mediated gene expression response to environmental stresses (signals) in plants. However, this hypothesis needs experimental validation in the future.

Similar to miR156, miR164 also responds to different stresses in diverse plants [4], [5], [10], [12], [22], [35], [36], [37]. By induction of an auxin signal, miR164 regulates molecular circuitry that controls the separation of developing organs [41] and normal flower development [42]. Auxin signal transduction is related to bacterial disease resistance in *Arabidopsis* [14]. We also noticed that poplar miR164 targeted some disease resistance proteins in this study. Therefore, miR164 might also play an important role in disease responsiveness in plants, especially in *Populus*; however, the resistance mechanism of miR164 (through auxin signal transduction, disease resistance proteins, or both pathways together) remains unclear.

### The TC-rich Repeat, W1-box, and MBS Motifs are Tightly Related to Disease Defense in *Populus*

Cis-acting regulatory elements are important molecular switches involved in the transcriptional regulation of a dynamic network of gene activities controlling various biological processes, such as the response to developmental processes, hormones, and abiotic and biotic stresses. To further elucidate the inducibility of miRNA-mediated gene regulation, *in silico* analysis of cis-acting elements in plant promoters have been used with success in studies of miRNA expression profiles [6], [10].

TC-rich repeats were the most abundant and ubiquitous cis-elements found in the present study. TC-rich repeats have been detected in the promoter region of many plant disease-resistance genes, e.g., defensin1 (products with antifungal properties) in *Picea glauca* [43], fungal elicitor-induced stilbene synthase in Chinese *Vitis pseudoreticulata* [44], [45], three key enzymes of the phenylpropanoid pathway in *Salvia miltiorrhiza* (phenylalanine ammonia-lyase (PAL), 4-coumarate:CoA ligase (4CL), and chalcone synthase (CHS) genes) [46]. TC-rich repeats were also identified in the target gene of rice miR396d [47] and poplar miR168a, miR395b, miR472a, and miR472b [10]. Therefore, we conclude that TC-rich repeats are involved in gene expression regulation of plant resistance to disease, at both the transcriptional and post-transcriptional level. However, the precise function and regulatory mechanism of TC-rich repeats needs further study.

The W1-box site, a well-studied W-box site, is the binding site for the WRKY TF family in plants. W-box motifs have been found in promoters of many defense and response genes such as genes coding for PR1, PR10, glutathione S-transferase [48], and stilbene synthase (an enzyme of phytoalexin biosynthesis) [49] genes. After fungal attack or fungal elicitor treatment, WRKY TFs induce the expression of plant defense or response genes by combining with the W-box motif. However, the prevalent W1-box presentation in fungal response miRNAs of poplar in this study revealed its another side of the defense function in response to disease. Initiated by fungal elicitors, WRKY TFs also decrease the expression of specific defense and response genes through negative regulation of miRNAs. That is, W-box motifs have two controversial effects on disease defense at the transcriptional and post-transcriptional level in plants. The arms race between these two diverse regulatory mechanisms might determine the level of plant resistance to diseases or its pathological development process.

MBS is the binding site for MYB TFs. The fact that MYB genes are widespread in plants (for example, 166 members in *Arabidopsis* and 213 in *Populus* (http://plntfdb.bio.uni-potsdam.de/v3.0)) suggests that they may play key roles in some basic biological processes. Some MYBs are involved in the regulation of cell proliferation, differentiation, and apoptosis [50], facilitate anther development [51], and determine the fate of plant cells [28]. MYBs are also thought to take part in the complex signaling network through which plants respond to changes in the surrounding environment [52], [53]. Although experimental evidence for MYB involvement in plant disease stress is scarce, the ubiquitous distribution of MBS motifs in this study and the three fungal response miRNA (miR159, miR164, and miR319) targeting MYB TFs illustrate that MBS motifs are also tightly associated with plant disease response.

Plants always regulate disease defense through phytohormone signal transduction [54], and several miRNAs (miR159, miR160, miR164, and miR167) were induced by phytohormones [55], [56]. Hence, the occurrence of hormone-related cis-elements (ERE, GARE, CGTCA motif, and ABRE) in the fungal response miRNAs implies that the pathway mediated by phytohormones is also crucial in the regulation of the miRNA-mediated gene expression of poplar canker.

In this study, more than one kind of stress-responsive cis-elements were found in the promoter region of miRNAs. This might be the main reason why our fungi-responsive miRNAs also appeared in other stress responses. In general, different kinds of cis-element present in the promoter region of one miRNA, TF, or protein-encoding gene might represent the molecular basis of cross-talk among different environmental stresses, as well as cross-talk among hormones, TFs, and miRNAs.

### The Feedback Regulatory Network of miRNA-TF Interaction in Poplar Canker

Using bioinformatics analysis, two feedback regulatory network of miR160-ARF (Auxin receptor factor) and miR167-ARF interaction was found in *Arabidopsis* [57]. Moreover, an auxin signal transduction feedback regulatory network of miR167-ARF in rice [58], [59] and miR164-NAC in *Arabidopsis* [60] have also been described. Auxin signal transduction is related to bacterial disease resistance in *Arabidopsis* [14], and this study indicated that miR160, miR167, and miR164 are tightly related to plant disease resistance. Other TF-related feedback regulatory networks are the miR156-SPL interaction in *Arabidopsis* [40], and miRNA-WRKY in rice, maize, and *Arabidopsis* [47], [56], [61]. In these diverse examples, miR156 and miR164, two miRNAs that respond to many environmental stresses, are both involved in the miRNA-TF feedback regulatory network, suggesting their key roles in stress responses.

**Figure 6:**
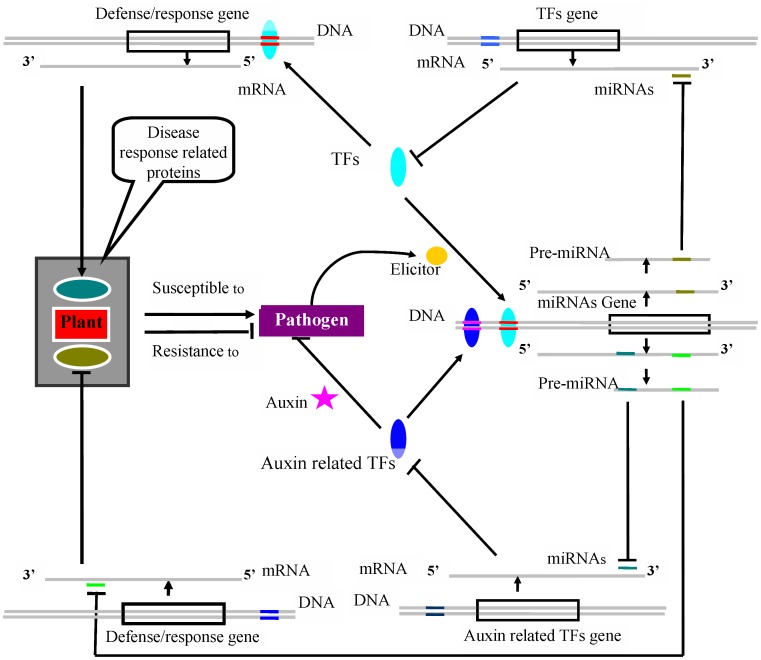
A putative miRNAs-TFs feedback regulatory model in plant-pathogen interaction. This model conducted mainly based on the feedback regulatory network of miRNAs-WRKY (or MYB) and miRNAs-ARF in plants. The mRNA levels of TFs were feedback regulated by some specific miRNAs. Defense/response genes positively regulated by TFs proteins at transcriptional level, while negatively regulated by miRNAs at post-transcriptional level. The symbol “→” and “□” represented induced and repressed respectively.

The miRNA-WRKY feedback network [62] was not found in this study, although W1-box motifs were found in 32 fungi-responsive miRNAs of *P. trichocarpa*. However, a novel miRNA-TF interactome was implied in this study. Poplar canker pathogen, *B. dothidea*, induced expression of miR159, miR164, and miR319, and these three miRNAs targeted MYB factors (Table S3), suggesting the miRNA-mediated regulation of MYB factors. The promoters of these three miRNAs are highly abundant in the MBS box, implicating MYB in their activation/repression; thus, evidence that miR159, miR164, and miR319 are positively regulated by the transcription factors they target suggests that the expression of these targets is modulated by a negative feedback loop that buffers small changes in the level of their mRNAs. Therefore, the feedback regulatory networks of miRNA-TF in *Arabidopsis* infected with *P. syringae*, the powdery mildew fungus *Golovinomyces orontii* [61], and in *Populus* with *B. dothidea* suggest that the miRNA-TF interactome is not only prevalent in phytohormone signaling pathways (auxin) but also in plant disease or stress responses. Based on the literature and the results of the present study, a feedback regulatory network of miRNA-TF in plant disease response is illustrated in Figure 6. This miRNA-TF feedback network might help fine-tune the gene expression regulation during pathogen attack or abiotic stresses in plants.

To the best of our knowledge, this is the first report on miRNA-mediated gene regulation after fungal pathogen attack in trees. As an extremely polyphagous fungal species, *B. dothidea* is also a common pathogen and reduces the growth and productivity of many ecological and/or economic tree species (e.g., apple, peach, prunes, peanut). Therefore, the present study should also shed light on the research and management of *B. dothidea*-related diseases in other trees.

## Materials and Methods

### Plant Material, Pathogen Inoculation, and Total RNA Extraction

Five-month-old *P. trichocarpa* clone M16 plantlets grown in a greenhouse at the Chinese Academy of Forestry (CAF, Beijing, China) were used for the experiment. Twelve plantlets were selected for inoculation and all trees were approximately 1.5 m tall and 7–9 mm in stem diameter.

One pathogenic fungal isolate, *B. dothidea* strain CZ060 (deposited in the College of Forestry at Beijing Forestry University), was isolated from poplar bark with canker symptoms in Baoding, Hebei Province, China. The fungal isolate was grown on 2% potato dextrose agar (PDA; 20 g potato extraction, 20 g dextrose, 15 g agar, 1 L water; Beijing Shuangxuan microbe culture medium products, Beijing, China) at 25°C in darkness, and used as inoculum.

For inoculations, wounds were made in the stems of the plants using a 4 mm diameter cork borer to remove the bark and expose the cambium. Five wounds were made on the stem of each poplar tree, from approximately 20 cm above soil level to the top, in 20 cm intervals. Plugs of fungal mycelium were taken from 7-day-old cultures grown on PDA using the same size cork borer, and were placed into the wounds, with the mycelium facing the cambium. Inoculated wounds were sealed with sterile polyethylene cling wrap to prevent desiccation and contamination. In this study, nine plantlets were inoculated with isolate CZ060 while the other three plantlets were inoculated with sterile PDA plugs to serve as controls. All inoculated and control poplar trees were cultured in the greenhouse.

After 3, 5, and 7 days of inoculation, barks (approximately 1 m from the base, except the petioles and buds) of three inoculated plants (biological replications) was removed from the trees, immediately mixed, and stored in liquid nitrogen. Bark of the three control poplars was removed 3 days after inoculation. To avoid contamination from fungal mycelium, bark from about 5 mm around the inoculation spot in both infected and control poplar was also removed.

Total RNA was extracted from poplar stem bark tissue using the standard CTAB method for plants [63]. The equal amount volume total RNA of three individual replications was mixed and used for microarray profiling, while the total RNA of three individual replications was used for RT-qPCR validation of miRNAs and their target genes.

### miRNA Microarrays

Affymetrix® GeneChip miRNA array analysis was conducted to detect the expression patterns of miRNAs induced by *B. dothidea* using total RNA (included small RNAs) by a service provider (Shanghai Biotechnology Corporation (SBC), Shanghai, China). The oligo-nucleotide hybridization graphs were scanned with the Affymetrix® scanner and detected with the software GCOS1.4. The hybridization data were analyzed with the miRNA QC tool using the default parameters. The genes with fold changes of ≥2 compared to controls were identified as differentially expressed genes. For a straightforward comparison of differences between samples, the expression profiles of the fungi-responsive miRNAs were analyzed using Gene clustering 3.0 [64] and visualized with Java Tree View [65].

### Real-time qPCR Validation of miRNA Expression

The reverse transcription and real-time qPCR reaction of mature miRNA genes were detected with the All-in-One™ miRNA qPCR detection kit (GeneCopoeia, Inc. USA). Each reverse transcription reaction consisted of 2 µg RNA, 2.5 U PolyA polymerase, 1µl RTase mix, and 1× reaction buffer in a final volume of 25 µl. The reaction mixtures were incubated at 37°C for 60 min, followed by a short centrifugation, incubated at 85°C for 5 min, and then diluted five times with sterile ddH_2_O.

Real-time qPCR was conducted with the Applied Biosystems 7500 HT sequence detection system (Applied Biosystems, Foster City, CA, USA). For each reaction, 2 µl diluted first-strand cDNA was mixed with 10 µl 2× All-in-One qPCR mix, 0.4 µl 50× ROX reference dye, 0.2 µM forward and reverse primer in a final volume of 20 µl. The amplification conditions were as follows: 40 cycles at 94°C for 10 s, 60°C for 20 s, and 72°C for 10 s. The amplification was followed by a thermal denaturing step to generate dissociation curves for verifying amplification specificity. The reverse primer was the All-in-One universal reverse adaptor PCR primer, while the forward primers were synthesized mainly according to the mature sequence of the tested miRNAs. However, for some miRNAs belonging to one miRNA family, only 15 specific primers were designed for testing the 41 miRNAs. For example, three miRNAs of the miR159 family (miR159a, miR159b, and miR159c) have the same mature sequence and use the same forward primer. In total, 15 qPCR validation reactions (designed for detecting the expression of miR156, miR159, miR160, miR164, miR166, miR168, miR172, miR319, miR398, miR408, miR1448, and miR1450) for the 41 fungi-response miRNAs tested were carried out. The miRNA validation qPCR reactions, mature miRNA sequences, and forward primers used in this study are listed in Table S5. The relative gene expression was calculated using the 2^−ΔΔC^_T_ method [66] and the *Populus* 5.8S rRNA (5′-GTCTGCCTGGGTGTCACGCAA-3′) [4] was used as an endogenous reference.

### Real-time PCR Validation of Target Gene Expression

Seven target genes for the differentially expressed miRNAs were validated using real-time qPCR. A total of 2 µg RNA was used for the reverse transcription reaction and qPCR with the Takara real-time qPCR detection kit (Takara, Dalian, China) according to the manufacturer’s instructions. The relative gene expression was also calculated using the 2^−ΔΔC^_T_ method [66], with the UBQ sequence as a reference. The primer sequences of target genes are listed in Table S6.

### Cis-acting Element Analysis of miRNA Genes Induced by *Botrosphaeria dothidea*

Each pre-miRNA sequence of the differentially expressed miRNAs was derived from miRBase release 18.0 (http://www.mirbase.org), and the upstream sequence of each pre-miRNA gene was derived from the JGI Poplar database (http://genome.jgi-psf.org/Poptr1_1/Poptr1_1.home.html). The upstream sequence (2500 bp in length) was used for the cis-acting element in PlantCARE (http://bioinformatics.psb.ugent.be/webtools/plantcare) [67].

## Supporting Information

Table S1 **The miRNA microarray data for** ***P.trichocarpa*** **after fungal inoculation.**
(DOC)

Table S2 **Real-time qPCR results for fungi-responsive miRNAs in** ***P.trichocarpa.***
(DOC)

Table S3 **Potential targets of the fungi-response miRNA in** ***Populus trichocarpa*** **.**
(DOC)

Table S4 **Expression of miRNAs targets inoculated by canker pathogen in** ***P.trichocarpa.***
(DOC)

Table S5 **Primers sequences used for real time qPCR analysis of fungi-response miRNAs.**
(DOC)

Table S6 **Primers sequences used for the validation of miRNA targets.**
(DOC)
