# Supplementary material for: Involvement of microRNA-Mediated Gene Expression Regulation in the Pathological Development of Stem Canker Disease in Populus trichocarpa
Source: PLoS One. 2012 Sep 18;7(9):e44968. doi: 10.1371/journal.pone.0044968 (PMC3445618; doi:10.1371/journal.pone.0044968)
Supplement: Table S1 — The miRNA microarray data for P.trichocarpa after fungal inoculation. (DOC) [file pone.0044968.s001.doc]

Table S1 The miRNA microarray data for *P.trichocarpa* after fungal inoculation.

| miRNAs | 3-DAI vs CK fold_change^1^ | 5-DAI vs CK fold_change^2^ | 7-DAI vs CK fold_change |
| --- | --- | --- | --- |
| miR156g | 1.11 | 1.97 | 2.60 |
| miR156h | -1.06^1^ | 2.22^2^ | 2.77 |
| miR156i | 1.08 | 2.10 | 2.95 |
| miR156j | -1.56 | 1.75 | 2.27 |
| miR159a | 12.80 | 7.98 | 12.10 |
| miR159b | 66.83 | 51.07 | 84.04 |
| miR159c | 22.38 | 13.13 | 22.23 |
| miR159d | 24.24 | 14.87 | 21.19 |
| miR160a | 1.09 | 1.39 | 2.06 |
| miR164a | 1.59 | -2.11 | -1.30 |
| miR164e | 1.26 | -2.07 | -1.40 |
| miR166a | 4.49 | 8.76 | 11.93 |
| miR166b | 5.75 | 10.10 | 12.79 |
| miR166c | 5.93 | 9.89 | 15.82 |
| miR166d | 3.38 | 8.43 | 12.57 |
| miR166e | 3.81 | 9.04 | 12.14 |
| miR166f | 6.15 | 7.56 | 11.87 |
| miR166g | 4.38 | 6.60 | 12.75 |
| miR166h | 6.35 | 12.89 | 15.99 |
| miR166i | 4.87 | 7.55 | 11.58 |
| miR166j | 3.49 | 6.71 | 11.12 |
| miR166k | 3.32 | 4.87 | 8.33 |
| miR166l | 3.96 | 8.25 | 11.41 |
| miR166m | 5.98 | 10.20 | 12.11 |
| miR166n | 1.12 | 4.30 | 6.44 |
| miR166o | 1.04 | 3.74 | 4.66 |
| miR166q | 1.23 | 3.76 | 5.39 |
| miR168a | 8.48 | 2.10 | 3.04 |
| miR168b | 6.02 | 2.13 | 3.06 |
| miR172h | -1.07 | 2.20 | 1.56 |
| miR319a | 2.11 | -1.97 | -1.56 |
| miR319b | 10.00 | 2.20 | 2.70 |
| miR319c | 3.02 | -1.41 | -1.00 |
| miR319d | 3.78 | -1.06 | 1.17 |
| miR319f | 2.49 | 2.52 | 2.60 |
| miR319g | 2.34 | 2.30 | 2.33 |
| miR319h | 2.63 | 2.41 | 2.41 |
| miR398c | 2.94 | 1.37 | 1.30 |
| miR408 | 3.36 | 1.92 | 3.35 |
| miR1448 | -4.42 | -3.84 | -3.44 |
| miR1450 | -1.17 | 3.53 | 4.07 |

Note: 1. Microarray data with a negative sign represented a down-regualtory expression, while the data without the negative sign represented an up-regulatory expression. 2. The yellow highlighted data represented a significant change (the fold change ≥2 was selected as the criterion) when compared with the control treatment.
