# Supplementary material for: Involvement of microRNA-Mediated Gene Expression Regulation in the Pathological Development of Stem Canker Disease in Populus trichocarpa
Source: PLoS One. 2012 Sep 18;7(9):e44968. doi: 10.1371/journal.pone.0044968 (PMC3445618; doi:10.1371/journal.pone.0044968)
Supplement: Table S2 — Real-time qPCR results for fungi-responsive miRNAs in P.trichocarpa. (DOC) [file pone.0044968.s002.doc]

Table S2 Real-time qPCR results for fungi-responsive miRNAs in *P.trichocarpa.*

| miRNAs gene | 3-DAI vs CK | | | 5-DAI vs CK | | | 7-DAI vs CK | | |
| --- | --- | --- | --- | --- | --- | --- | --- | --- | --- |
|  | mean | SD | CV | Mean | SD | CV | Mean | SD | CV |
| miR156G | 2.68 | 0.31 | 0.12 | 4.44 | 0.58 | 0.13 | 3.47 | 0.12 | 0.04 |
| miR172H | 1.04 | 0.17 | 0.16 | 2.50 | 0.18 | 0.07 | 3.22 | 0.45 | 0.14 |
| miR159A | 2.27 | 0.17 | 0.07 | 4.51 | 0.33 | 0.07 | 6.27 | 0.42 | 0.07 |
| miR319F | 1.19 | 0.16 | 0.14 | 1.94 | 0.20 | 0.10 | 2.41 | 0.25 | 0.10 |
| miR159D | 1.39 | 0.24 | 0.17 | 1.67 | 0.20 | 0.12 | 2.56 | 0.31 | 0.12 |
| miR398C | 0.88 | 0.17 | 0.31 | 9.93 | 1.33 | 0.13 | 6.67 | 0.58 | 0.09 |
| miR160A | 0.99 | 0.17 | 0.18 | 3.29 | 0.17 | 0.05 | 1.87 | 0.16 | 0.09 |
| miR408 | 1.05 | 0.17 | 0.16 | 10.73 | 0.90 | 0.08 | 3.66 | 0.21 | 0.06 |
| miR164A | 4.93 | 0.47 | 0.09 | 8.54 | 1.01 | 0.12 | 6.11 | 0.70 | 0.12 |
| miR1450 | 1.81 | 0.10 | 0.05 | 2.58 | 0.27 | 0.11 | 3.56 | 0.21 | 0.06 |
| miR166A | 1.75 | 0.13 | 0.08 | 2.23 | 0.23 | 0.10 | 3.93 | 0.48 | 0.12 |
| miR166N | 1.85 | 0.37 | 0.20 | 2.10 | 0.22 | 0.10 | 1.81 | 0.28 | 0.15 |
| miR168A | 1.75 | 0.12 | 0.07 | 2.28 | 0.32 | 0.14 | 2.89 | 0.19 | 0.07 |
| miR1448 | 1.25 | 0.18 | 0.14 | 3.81 | 0.37 | 0.10 | 1.72 | 0.28 | 0.16 |
