# Supplementary material for: Involvement of microRNA-Mediated Gene Expression Regulation in the Pathological Development of Stem Canker Disease in Populus trichocarpa
Source: PLoS One. 2012 Sep 18;7(9):e44968. doi: 10.1371/journal.pone.0044968 (PMC3445618; doi:10.1371/journal.pone.0044968)
Supplement: Table S3 — Potential targets of the fungi-response miRNA in Populus trichocarpa . (DOC) [file pone.0044968.s003.doc]

Table S3 Potential targets of the fungi-response miRNA in *Populus trichocarpa*.

| MiRNAs | Function annotations | Predicted target genes (from Lu et al. 2005 and Lu et al. 2008) |
| --- | --- | --- |
| miR156 | cationic amino acid transporter | fgenesh4_pm.C_LG_XII000479 |
|  | disease resistance protein (NBS-LRR class) | estExt_fgenesh4_pg.C_4370006, eugene3.00640226,fgenesh4_pg.C_scaffold_10484000001, fgenesh4_pg.C_scaffold_1683000002 |
|  | nitrate transporter | grail3.0015018701 |
|  | squamosa promoter-binding protein | estExt_Genewise1_v1.C_1240186, estExt_Genewise1_v1.C_LG_XV2187,eugene3.00120942, eugene3.00160416, fgenesh4_pg.C_LG_II001303,  fgenesh4_pg.C_LG_X001404 |
|  | unknown | fgenesh4_pg.C_LG_VII000454, fgenesh4_pg.C_scaffold_9189000001, grail3.0008008901, grail3.0010026801 |
| miR159 | Myb | fgenesh4_pm.C_scaffold_40000020, eugene3.00091462, gw1.I.6885.1, gw1.III.41.1, |
|  | peroxidase 21 precursor | eugene3.00280149 |
|  | (1-4)-beta-mannan endohydrolase | eugene3.00161335 |
|  | cytokinin oxidase family protein | eugene3.00060432, gw1.XVI.1482.1 |
|  | TCP family transcription factor | eugene3.00190830, eugene3.00110429 |
|  | H+-exporting ATPase | fgenesh4_pg.C_LG_III000605 |
|  | sexual differentiation process protein | fgenesh4_pg.C_LG_I002904, gw1.I.4838.1 |
|  | haloacid dehalogenase-like hydrolase family protein | gw1.XIII.601.1 |
|  | PPR | fgenesh4_pg.C_LG_XIV000041 |
|  | leucine rich repeat family protein | gw1.V.3546.1, gw1.V.1354.1 |
|  | protein kinase | fgenesh4_pm.C_LG_II000238 |
|  | DnaJ family protein | eugene3.00190756 |
|  | reticulon family protein | estExt_Genewise1_v1.C_LG_VII0576 |
|  | unknown | fgenesh4_pg.C_LG_X001415, eugene3.00111000, fgenesh4_pg.C_scaffold_134000031, gw1.V.1257.1, eugene3.00012728, fgenesh4_pg.C_LG_I000129, gw1.XIII.3464.1, eugene3.00090112, eugene3.01420013, eugene3.00121020 |
| miR160 | ARF | eugene3.00660262, estExt_fgenesh4_pm.C_LG_X0888, fgenesh4_pg.C_LG_IX001411,  fgenesh4_pg.C_LG_VIII000301, estExt_fgenesh4_pm.C_LG_XVI0323,  fgenesh4_pg.C_LG_II000830, gw1.28.631.1, gw1.28.632.1, estExt_fgenesh4_pg.C_LG_V0901, eugene3.00640087 |
| miR164 | NAC domain protein | gw1.V.3536.1, gw1.VII.2722.1, fgenesh4_pm.C_LG_XII000069, eugene3.00150202 |
|  | leucine rich repeat family protein | eugene3.00110658 |
|  | transporter-related | gw1.164.27.1 |
|  | TMV resistance protein-like | estExt_Genewise1_v1.C_LG_II2421 |
|  | Myb | estExt_Genewise1_v1.C_LG_VI0902 |
|  | unknown | eugene3.00030331 |
| miR166 | homeodomain-leucine zipper protein | fgenesh4_pm.C_LG_I000560, estExt_fgenesh4_pg.C_LG_III0436, estExt_Genewise1_v1.C_660759,  gw1.6326.1.1, fgenesh4_pg.C_LG_XVIII000250, estExt_fgenesh4_pm.C_LG_VI0713, gw1.IX.4748.1, estExt_fgenesh4_pg.C_LG_I2905 |
|  | NAC domain protein | fgenesh1_pg.C_LG_XIII001042 |
| miR168 | coatomer alpha subunit-like protein | eugene3.00120688, eugene3.00150490 |
|  | argonaute (AGO1) | grail3.0122002801 |
|  | unknown | grail3.0110002401, fgenesh4_pg.C_LG_III001280 |
| miR172 | Homeotic protein APETALA2 | grail3.0019003502, eugene3.00050501,fgenesh1_pg.C_LG_X001967,fgenesh1_pg.C_scaffold_28000114,  eugene3.00160775 |
|  | unknown | estExt_fgenesh1_pg_v1.C_LG_VIII1617 |
| miR319 | Myb | grail3.0100003501 |
|  | unknown | eugene3.00131146, eugene3.00090112 |
| miR398 | selenium-binding protein | estExt_Genewise1_v1.C_LG_I3319 |
|  | Cu2+/Zn2+ superoxide dismutase | estExt_Genewise1_v1.C_LG_XIII1233 |
|  | arsenate reductase | eugene3.146400001 |
| miR1448 | Disease resistance protein | eugene3.01310091, eugene3.00190077 |
|  | Glutathione S-conjugate ABC transporter (MRP2) | gw1.1700.5.1 |
|  | ATP-binding cassette transport protein | gw1.IV.2236.1 |
|  | Unknown | fgenesh4_pg.C_LG_V000530 |
| miR1450 | Leucine-rich repeat transmembrane protein kinase | eugene3.00141443 |
|  | Unknown | fgenesh4_pg.C_LG_V001134, fgenesh4_pg.C_LG_XII001210 |
| miR408 | plastocyanin-like | estExt_fgenesh4_pm.C_LG_II1118, estExt_fgenesh4_pg.C_LG_I1252 |
|  | GTP-binding protein | gw1.VI.478.1, eugene3.114030001, eugene3.00180337 |
|  | phytoalexin-deficient 4 protein | gw1.VII.1589.1 |
|  | laccase | eugene3.00191007 |
|  | SYN1 splice variant 1 | fgenesh4_pg.C_LG_VIII000625 |
