# Supplementary material for: Involvement of microRNA-Mediated Gene Expression Regulation in the Pathological Development of Stem Canker Disease in Populus trichocarpa
Source: PLoS One. 2012 Sep 18;7(9):e44968. doi: 10.1371/journal.pone.0044968 (PMC3445618; doi:10.1371/journal.pone.0044968)
Supplement: Table S4 — Expression of miRNAs targets inoculated by canker pathogen in P.trichocarpa. (DOC) [file pone.0044968.s004.doc]

Table S4 Expression of miRNAs targets inoculated by canker pathogen in *P.trichocarpa.*

| Target genes, gene model (miRNAs genes) | 3-DAI vs CK | | | | 5-DAI vs CK | | | | 7-DAI vs CK | | | |
| --- | --- | --- | --- | --- | --- | --- | --- | --- | --- | --- | --- | --- |
|  | Test 1 | | Test 2 | | Test 1 | | Test 2 | | Test 1 | | Test 2 | |
|  | Mean | SD | Mean | SD | Mean | SD | Mean | SD | Mean | SD | Mean | SD |
| POD, eugene3.00280149 (miR159) | 0.13 | 0.05 | 0.21 | 0.05 | 0.16 | 0.03 | 0.33 | 0.07 | 0.13 | 0.03 | 0.18 | 0.04 |
| NBS-LRR, eugene3.00190077 (miR1448) | 0.60 | 0.14 | 0.42 | 0.19 | 0.44 | 0.12 | 0.30 | 0.02 | 0.32 | 0.05 | 0.39 | 0.09 |
| LRR transmembrane protein, eugene3.00141443 (miR1450) | 0.86 | 0.19 | 0.85 | 0.39 | 0.52 | 0.12 | 0.37 | 0.08 | 0.84 | 0.15 | 0.67 | 0.20 |
| CKX, gw1.XVI.1482.1 (miR159) | 0.73 | 0.21 | 0.52 | 0.12 | 0.89 | 0.21 | 0.71 | 0.24 | 0.27 | 0.05 | 0.33 | 0.11 |
| CSD, estExt_Genewise1_v1.C_LG_XIII1233 (miR398) | 0.27 | 0.07 | 0.32 | 0.08 | 0.27 | 0.08 | 0.21 | 0.05 | 0.42 | 0.25 | 0.59 | 0.21 |
| LRR, gw1.V.3546.1 (miR159) | 0.88 | 0.17 | 0.87 | 0.29 | 0.57 | 0.19 | 0.74 | 0.16 | 0.43 | 0.13 | 0.56 | 0.21 |
| HD-ZIP, estExt_fgenesh4_pg.C_LG_III0436 (miR166) | 1.07 | 0.17 | 0.90 | 0.11 | 0.32 | 0.11 | 0.43 | 0.09 | 0.88 | 0.06 | 0.65 | 0.12 |
| Plastocyanin-like protein,  estExt_fgenesh4_pg.C_LG_I1252  (miR408) | 0.25 | 0.15 | 0.14 | 0.02 | 0.69 | 0.30 | 0.64 | 0.15 | 0.55 | 0.16 | 0.40 | 0.04 |
| LRR, eugene3.00110658(miR164) | 0.67 | 0.14 | 0.62 | 0.04 | 0.87 | 0.29 | 0.69 | 0.05 | 0.68 | 0.14 | 0.73 | 0.19 |
