# Supplementary material for: Involvement of microRNA-Mediated Gene Expression Regulation in the Pathological Development of Stem Canker Disease in Populus trichocarpa
Source: PLoS One. 2012 Sep 18;7(9):e44968. doi: 10.1371/journal.pone.0044968 (PMC3445618; doi:10.1371/journal.pone.0044968)
Supplement: Table S5 — Primers sequences used for real time qPCR analysis of fungi-response miRNAs. (DOC) [file pone.0044968.s005.doc]

Table S5 Primers sequences used for real time qPCR analysis of fungi-response miRNAs.

| PCR reactions | miRNAs molecules detected^1^ | mature miRNAs sequence^2^ |
| --- | --- | --- |
| miR156G | miR156g, h,i,j | TTGACAGAAGATAGAGAGCAC |
| miR159A | miR159a,b,c | TTTGGATTGAAGGGAGCTCTA |
| miR159D | miR159d | CTTGGATTGAAGGGAGCTCCT |
| miR160A | miR160a | TGCCTGGCTCCCTGTATGCCA |
| miR164A | miR164a,e | TGGAGAAGCAGGGCACGTGCA |
| miR166A | miR166a,b,c,d,e,f,g,h,I,j,k,l,m | TCGGACCAGGCTTCATTCCCC |
| miR166N | miR166n,o,q | TCGGACCAGGCTTCATTCCTT |
| miR168A | miR168a,b | TCGCTTGGTGCAGGTCGGGAA |
| miR172H | miR172h | GGAATCTTGATGATGCTGCAG |
| miR319A | miR319a,b,c,d | TTGGACTGAAGGGAGCTCCC |
| miR319F | miR319f,g,h | TTGGACTGAAGGGAGCTCCT |
| miR398C | miR398c | TGTGTTCTCAGGTCGCCCCTG |
| miR408 | miR408 | ATGCACTGCCTCTTCCCTGGC |
| miR1448 | miR1448 | CTTTCCAACGCCTCCCATAC |
| miR1450 | miR1450 | TTCAATGGCTCGGTCAGGTTAC |
| Ptc 5.8S |  | GTCTGCCTGGGTGTCACGCAA |

Note: 1. miRNAs molecules that detected in one PCR reaction. For example, four miR156 molecules (miR156g, miR156h, miR156i and miR156j) were detected in one qPCR reaction (miR156G) with the same forward primer and the universal GeneCopoeia miRNA reverse primer. 2. The sequence underlined were the forward primers used in real time qPCR analysis.
