# Supplementary material for: Involvement of microRNA-Mediated Gene Expression Regulation in the Pathological Development of Stem Canker Disease in Populus trichocarpa
Source: PLoS One. 2012 Sep 18;7(9):e44968. doi: 10.1371/journal.pone.0044968 (PMC3445618; doi:10.1371/journal.pone.0044968)
Supplement: Table S6 — Primers sequences used for the validation of miRNA targets. (DOC) [file pone.0044968.s006.doc]

Table S6 Primers sequences used for the validation of miRNA targets.

| Gene model of miRNA target gene in JGI *Populus* genome release 1.1 | Gene model of miRNA target gene in JGI *Populus* genome release 2.0 | MiRNA  family | Forward primers | Reverse primers |
| --- | --- | --- | --- | --- |
| eugene3.00280149 | POPTR_0006s13190.1 | miR159 | ACGGCATTGTCATGTTAGGAG | TCAGGGTCCATAGTTGGATCA |
| gw1.XVI.1482.1 | POPTR_0016s04340.1 | miR159 | GTCAACAAGTCCAAGTGGAACA | ATTCTTCCTGGGTGCTGTAATG |
| gw1.V.3546.1 | POPTR_0005s10140.1 | miR159 | AAGCTAGTCAAGATGGGGAGAAG | CCAGTGTAACCAAACTCGATAGC |
| estExt_fgenesh4_pg.C_LG_III0436 | POPTR_0003s04860.1 | miR166 | TGTTGGCCATTCTCCTGAA | GGCTTCCTTCCCAGAATCA |
| estExt_Genewise1_v1.C_LG_XIII1233 | POPTR_0013s03160.1 | miR398 | GAAGCGGCCAGAAACATCTA | TTGGACCATCTCCTTCTTGG |
| eugene3.00190077 | POPTR_0019s00620.1 | miR1448 | AGGCATGGTTAAAGCAGGTG | ACCTTGGTGAATGGCTTGAG |
| eugene3.00141443 | POPTR_0014s18490.1 | miR1450 | ACACCAATCTGGGAAAGCTG | ATCGAACCAATGCTTTCAGG |
| eugene3.00110658 | POPTR_0122s00210.1 | miR164 | GAAGGTGGTTTTGGTCCTGT | TCAGGTAATTGACCCTGCAC |
| estExt_fgenesh4_pg.C_LG_I1252 | POPTR_0001s21660.1 | miR408 | CTTCAATGTTTCTGGCTGGC | TTTCACGCAGCATTAACAGC |
| UBQ (Control gene) |  |  | GTTGATTTTTGCTGGGAAGC | GATCTTGGCCTTCACGTTGT |
